# Engaging high school students in neuroscience research -through an e-internship program

**DOI:** 10.12688/f1000research.10570.2

**Published:** 2017-03-29

**Authors:** Wim E Crusio, Cynthia Rubino, Anna Delprato

**Affiliations:** 1 BioScience Project, Wakefield, USA; 2 University of Bordeaux, Institut de Neurosciences Cognitives et Intégratives d'Aquitaine (UMR 5287), Pessac Cedex, France; 3 CNRS, Institut de Neurosciences Cognitives et Intégratives d'Aquitaine (UMR 5287), Pessac Cedex, France

**Keywords:** e-internship, personalized learning, neurogenomics, systems biology, K12, stem

## Abstract

In this article, we describe the design and implementation of an e-internship program that BioScience Project offers high school students over the summer. Project topics are in the areas of behavioral neuroscience and brain disorders. All research, teaching, and communication is done online using open access databases and webtools, a learning management system, and Google apps. Students conduct all aspects of a research project from formulating a question to collecting and analyzing the data, to presenting their results in the form of a scientific poster. Results from a pilot study involving fifteen students indicate that students are capable of successfully completing such a project, and benefit both intellectually and professionally from participating in the e-internship program.

## Introduction

Neurogenomics is the study of the systems, networks, and gene interactions that underlie neural processes. Increased functional information from diverse sources available in open access databases, along with specific tools for analysis, enables the integration of these data to gain unique insights ( Overall *et al*., 2015). BioScience Project ( www.bioscienceproject.org) offers high school students the opportunity to work as summer interns on research projects in the area of behavioral neuroscience and brain disorders, which includes the analysis of gene expression data using systems and network biology methods (see Schughart & Williams, 2017). This is a voluntary internship available to students regardless of academic performance or institution. Students participate in the program to gain hands on experience and acquire new skills. The projects involve learning how to formulate and test hypotheses, data-mine biological and neuroscience-specific databases, statistical analysis, and data representation and visualization. Students need only a computer with an Internet connection to participate. The projects are flexible, allowing students to work from home on their own schedule. All communication is done via the Internet with an online learning management system (Moodle, https://moodle.com/), Google apps ( https://gsuite.google.com/), and video conferencing. At the end of the internship, students communicate their work in a poster, which can be used to leverage their college applications and/or detail their experience to prospective employers. Students also receive certificates of completion. Several strengths of the e-internship program worth noting are: (1) Students are highly interested in topics related to behavioral neuroscience and brain disease; (2) This is shown to be an effective model to introduce early stage students to advanced topics and research methods in neuroscience; (3) Students receive the otherwise-limited opportunity to participate in authentic research projects and work directly with professional scientists; (4) The internship program is scalable, enabling many students to participate; (5) Project results are freely accessible to the scientific community on BioScience Project’s website ( www.bioscienceproject.org).

Science outreach programs that connect professional scientists to students and teachers such as “Scientist in the Classroom” ( Laursen *et al*., 2007), “Shadow a Scientist” ( Clark *et al*., 2016) and the “Virtual Scientist” ( McCombs *et al*., 2007) report enhanced student interest in science and an increase in the understanding of science concepts and relevance to real life. The experience also provides an awareness of ongoing research and current methods. Internships bridge theory and practice and are a useful way to supplement and enrich an individual’s educational experience ( Ruggiero & Boehm, 2016). The e-internship model extends this opportunity to a broader group of students.

## Internship implementation

### Recruitment

Recruiting students is mainly done by contacting high school science departments through email and providing information about our organization and the internship opportunity. We include a recruitment poster ( Supplementary File 1) and ask that the information be passed along to their students. We launched a two year pilot project that included both private and public institutions around the Boston (MA, USA) area. Schools were selected randomly. Several students from schools not contacted by us learned about the Internship program through word of mouth or an Internet search.

### Internship design

The internship program runs for 6 to 8 weeks in July and August. Students may begin sooner if they like. The time commitment varies for each student, but is in the range of 10–15 hours per week. Students proceed at their own pace and can work alone or in a group. There are no deadlines, except to finish projects before the new school year begins. Project completion requires that students profile gene expression data associated with a brain disorder or behavior using a protocol designed by our laboratory and make a scientific-style poster of their work, which includes introduction, methods, results, and discussion sections.

Students are able to choose their topic of study or can select from subjects suggested by us. Project specific materials are provided throughout the internship. These include relevant literature for background information from science magazines ( *Scientific American* and *The Scientist*), as well as links to news updates from sources such as EurekAlert! ( https://www.eurekalert.org/), BBC Science ( http://www.bbc.co.uk/science), Neuroscience News ( http://neurosciencenews.com/) and YouTube ( https://www.youtube.com/).

Students are provided with as much mentoring as they need to complete the internship. Mentoring sessions are done through video conferencing and consist of a walk-through of each database with screen sharing, project discussion, and troubleshooting of problems. Mentoring sessions typically take place weekly and last anywhere from 30 to 90 min. Mentoring is requested and scheduled by email. All instruction and mentoring is provided by the project director, Dr. Anna Delprato. As the internship program grows, additional scientists will be recruited to assist with teaching. Students are not tested and there are no grades assigned. Teaching and communication is done through an online learning platform (Moodle; https://moodle.com/), one on one video conferencing (Skype or Google Hangouts), email, document sharing (Google Docs), and a Google group, which enables students to receive notices and communicate with one another. Google apps are also used for data handling (Google Sheets) and presentation (Google Slides). Students may also use Microsoft Office’s Excel and PowerPoint software for the same purpose.

### Project design

The protocol used for profiling gene expression data is broken down into steps so as not to overwhelm the students with too much information at once. Modules which consist of detailed instruction are built around each step of the protocol. The purpose of the module is to provide the students with a detailed reference in addition to the mentoring sessions so that they can work in the databases and with the data independently.

The starting point for all projects involves the identification of brain regions associated with a behavioral process or brain disease, which is based primarily on functional magnetic resonance imaging (fMRI) data. Students find this information through an Internet search with our assistance. Gene expression patterns are then analyzed to identify those genes that are preferentially expressed in these brain areas across all donor brains. For the genes identified in this way, clustering algorithms and gene ontology annotation are used to identify those entries that are directly related to the subject of interest. These genes are then used as hooks to build interaction networks in order to pull out additional functionally relevant genes.

All of the databases and analysis tools are open access. The core set of databases and web tools used in the internship are: The Allen Brain Atlas (gene expression data based on donor brains and correlation analysis; http://brain-map.org/), Venny (Venn diagram generator; http://bioinfogp.cnb.csic.es/tools/venny/), DAVID (Database for Annotation, Visualization and Integrated Discovery; functional annotation, pathway information, and clustering; https://david.ncifcrf.gov/; Huang *et al*., 2009), PythonAnywhere (statistics, graphing; https://www.pythonanywhere.com) and STRING (network analysis; http://string-db.org/; Szklarczyk *et al*., 2015). A more detailed description of these are provided in the following sections.

### Allen Brain Atlas/Gene Expression

The Allen Brain Atlas combines genomic data with neuroanatomy through the generation of gene expression maps obtained from Affymetrix data ( Hawrylycz *et al*., 2012). The Allen Brain human database contains gene expression data for 6 donor brains. This human database is queried using the differential search function, which enables a search to identify gene expression enrichment in one brain region as compared to another. For example, learning and memory are typically associated with the hippocampus, so in this case the differential search function is used to find genes that have enhanced expression in the hippocampus relative to other regions of the brain. Details on the usage of the differential search function can be found at the Allen Brain site ( http://help.brain-map.org/display/humanbrain/Microarray+Data#MicroarrayData-GeneSearch). Students are taught how to interpret Affymetix heatmaps, evaluate gene expression data (fold difference values, error, and threshold cutoff), and use spreadsheet editing, sorting, and graphing functions for the organization and analysis of large datasets.

The cleaned gene sets are then compared by the students to detect common and distinct elements using an online program (Venny) that evaluates lists and generates a Venn diagram as a visual representation. The genes that are common among all donors are then analyzed in DAVID for functional annotation, clustering, and pathway information. Genes that are associated with project relevant themes, such as behavior, nervous system development, and/or specific diseases, are used to build interaction networks, which consist of protein-protein interactions that are supported by multiple lines of evidence, such as experimental, text mining, and co-expression in the STRING database.

The interaction networks are used to identify potential gene candidates that may be involved in the same behavioral process or disease, and are also used to identify network substructures, such as hubs and motifs, which indicate important and possibly functionally related entities. Functional classification is assessed using DAVID to identify interactions that are relevant to the project topic. For an extended analysis, students can use the most pertinent genes extracted from the networks to identify additional candidates that have similar spatial expression profiles in the brain tissue of interest. The correlation analysis is done in the Allen Brain database using the correlation search function ( http://help.brain-map.org/display/humanbrain/Microarray+Data#MicroarrayData-CorrelativeSearch).

Finally, a statistical analysis of the gene expression data is performed by the students with Python, using an online Python server, PythonAnywhere, which enables students to run Python scripts from their browser. Students are provided with a general script and are required to modify this for their own datasets. The script returns general statistics, such as standard error, mean, minimum and maximum, variance, and distribution profiles.

## Internship outcomes

The internship program has run for two years since 2015. The first year, five students participated and in the second year ten students participated. Student project topics included addiction, learning and memory, Alzheimer’s disease, creativity, and bipolar disorder, among others. Student posters can be viewed at the BioScience Project website ( http://www.bioscienceproject.org/student-posters). This year a student also coauthored a published research article with our group, which reports on the identification of genetic factors associated with morphine addiction ( Crusio *et al*., 2016). Upon completion of the internship, students answered survey questions pertaining to the internship content, instruction, and overall experience. The student responses to the survey questions are presented in Table 1 and Table 2. Comments and suggestions provided by the students can be viewed in Supplementary File 2. Based on the student feedback in 2015, the internship instruction was revised for clarity using step-by-step annotated screenshots together with one-on-one tutorials via video conferencing for each database and method.

**Table 1.** Neuroinformatics Internship Student Survey 2015.

Survey Questions	Responses		
	Yes	No	Other
1. Was the internship content clearly presented?	4	1	
2. Was the internship content interesting?	5		
3. Could you navigate the module easily?	4		1 ^*^
4. Did you acquire new skills and knowledge?	5		
5. Would you recommend this internship to others?	5		
	Excellent	Good	Satisfactory
6. What overall rating would you give this internship?	2	2	1
7. What overall rating would you give the instructor?	4	1	

*“It was a bit confusing in the very beginning, but after a bit of experience it became very easy to use.”

**Table 2.** Neuroinformatics Internship Student Survey 2016.

Survey Questions	Responses		
	Yes	No	Other
1. Was the internship content clearly presented?	8	2 ^*^	
2. Was the internship content interesting?	10		
3. Could you navigate the module easily?	10		
4. Did you acquire new skills and knowledge?	10		
5. Would you recommend this internship to others?	10		
	Excellent	Good	Satisfactory
6. What overall rating would you give this internship?	4	5	1
7. What overall rating would you give the instructor?	10		

* Comment1: “It varied from step to step, sometimes it was clear and sometimes I was a little confused.” Comment2: “The steps were clearly presented and we had a lot of help through the ones we didn’t understand, but the overarching goal/conceptual understanding of the project was a bit confusing during the steps.”

## Conclusions

This e-internship program has been shown to be a useful way of introducing early stage students to advanced topics and research methods in systems biology, which supplements their high school science curriculum and provides them with an opportunity to gain hands on experience. Students are interested in collaborating with scientists on research projects in neuroscience-related topics and they gain both intellectually and professionally from participating in the summer e-internship program. Given the flexibility in both time and procedure, this e-internship program can easily be extended to include more students.

## Supplementary material

Supplementary File 1: Internship recruitment poster.

.

Supplementary File 2: Student feedback.
