# Supplementary material for: Engaging high school students in neuroscience research -through an e-internship program
Source: F1000Res. 2017 Mar 29;6:20. Originally published 2017 Jan 9. [Version 2] doi: 10.12688/f1000research.10570.2 (PMC5302143; doi:10.12688/f1000research.10570.2)

BioScience Project
Summer e-Internships in Neuroscience research

Students:
Intern with BioScience Project this summer. You will learn how to: design a research project, use biological databases and webtools, mine/analyze data, and prepare a scientific communication while working from home on your own schedule.

What you will gain:
A foundation for a prospective career, transferable skills, work experience to add to your resume, intellectual growth, an opportunity to work with professional scientists.

What you need to participate:
A 4-week commitment (~ 10 hrs/week), access to a computer with an internet connection. All students are welcome to participate in our internship program regardless of experience and academic performance.

About
BioScience Project is a non-profit research and teaching laboratory committed to the continuation of biomedical research and science education.

For more information please contact:
Anna Delprato, PhD
email: delprato@bioscienceproject.org
tel : (877) 339-5703
www.bioscienceproject.org

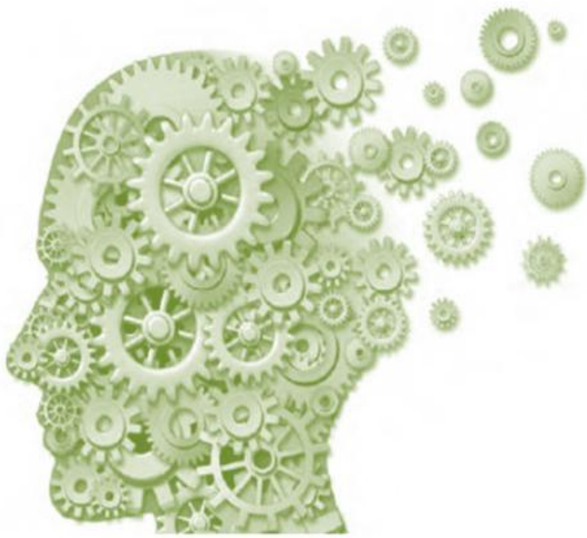

Supplement: Supplementary file 1 [file f1000research-6-12180-s0000.tgz › 7d3c6bfb-ff8c-4bc8-8c0a-14618c368665.pdf]
