# Supplementary material for: Engaging high school students in neuroscience research -through an e-internship program
Source: F1000Res. 2017 Mar 29;6:20. Originally published 2017 Jan 9. [Version 2] doi: 10.12688/f1000research.10570.2 (PMC5302143; doi:10.12688/f1000research.10570.2)
Supplement: Supplementary file 2 [file f1000research-6-12180-s0001.tgz › a86cbac6-3fc7-450c-b2c6-208bb77ba23e.docx]

**Supplementary File 2: Student feedback**

Student comments 2015

“Providing more information about what exactly the internship is and what is expected of the student before starting the internship would be helpful.”

“It would be helpful if there were some image tutorials for how to use some of the databases such as GOrilla (GOrilla database used in 2015 but not 2016) and STRING.”

“I really enjoyed the internship and learned a lot. The only thing is that I wish there was a clearer timeline for the project so I could have better managed my time.”

“Thanks for providing me with an opportunity to learn something new this summer!”

“Thank you so much for the opportunity. It was great and such an accomplishment. It really locked in that I want to pursue science specifically research as a career.”

“Thanks for giving me the opportunity to work with you! It was really an enjoyable and educational experience for me and will be a great help for me the future. It was really interesting using all of the databases. I think this internship is an exceptionally great experience because it allows a very flexible work schedule, both in time and in procedure, and I believe that the best work of an individual is only brought out when they have the freedom to do what they want.”

Student comments 2016

“Acquired amazing knowledge in the research of neuroscience. I also acquired technical skills especially involving the usage of bioinformatic tools and statistical analysis throughout the course of the bioscience project.”

“This internship allowed me to acquire skills and knowledge of bioinformatics that I don’t believe someone my age would have been able to acquire anywhere else!”
