# Supplementary material for: Engaging high school students in neuroscience research -through an e-internship program
Source: F1000Res. 2017 Mar 29;6:20. Originally published 2017 Jan 9. [Version 2] doi: 10.12688/f1000research.10570.2 (PMC5302143; doi:10.12688/f1000research.10570.2)
Supplement: Peer review history

# Peer review: Referee response for version 2

**DOI:** 10.5256/f1000research.12180.r21901

**Published:** 2017-04-18

**Authors:** Jennifer A. Ufnar

**Affiliations:** 1 Department of Teaching and Learning, Vanderbilt University, Nashville, TN, USA

I have read this submission. I believe that I have an appropriate level of expertise to confirm that it is of an acceptable scientific standard.

# Peer review: Referee response for version 1

**DOI:** 10.5256/f1000research.11391.r19554

**Published:** 2017-02-20

**Authors:** Jennifer A. Ufnar

**Affiliations:** 1 Department of Teaching and Learning, Vanderbilt University, Nashville, TN, USA

The article by Anna Delprato describes an interesting way to engage students in an online research program in the neurosciences. This reviewer is interested in the program and the potential impacts and uses of the program for K-12 students. This reviewer, however, has several concerns that need to be addressed before the paper is indexed.

  

1. The authors stipulate in the abstract that the students in the program were "capable of comprehending ...such a project". The authors cannot make that claim with the evidence presented in the manuscript. Self-reported surveys were the only form of data presented, and this type of survey does not show student learning or comprehension. The reader also does not know if this comprehension is applied to the ability to perform research, the content learned, or learning how to communicate the information (or a combination of all three). This is a significant concern and must be addressed before publication. 
2. There were only six references cited for the entire manuscript. There are many papers in the literature that discuss programs similar in nature to this. It would behoove the authors to include more background research to support their claims.
3. The authors need to include more information about the project itself (i.e. a discussion of the modules used for teaching, the mentoring, etc.). The reader is left wondering if the students are doing actually self-initiated projects, or if they are being led by the modules. It was helpful to read about the databases that the students use, but the reader does not gain any knowledge about the actual e-internship program through which the students progress (or are those the modules? It is unclear.). In other words, how were they mentored? Is the mentoring effective for student growth? How are the modules designed? Is there any research to show that the module design is effective? Do they present and defend their research in a scientific forum (even online)? It would also help to put the Project Design section first (before the database discussion).
4. The evaluation of the program needs to be more robust. Self-reported surveys are a good starting point, but they are only formative assessments. You need to include some summative assessments for the program and more qualitative assessments to determine the efficacy of the program for student learning. Also, it sounds as though the students are spending much less time on the project as opposed to an onsite laboratory model, so the reader is left wondering if the program is really effective. Also, do you have demographics of the participating students?
5. There are a couple of grammatical errors (mostly words that need to be omitted and comma usage errors). 

In summary, this manuscript presents an interesting program, but the authors need to address the issues of a clear evaluation plan to determine the efficacy of the program, and provide enough discussion of the program so that readers understand exactly what the students gain.

I have read this submission. I believe that I have an appropriate level of expertise to confirm that it is of an acceptable scientific standard, however I have significant reservations, as outlined above.

# Response

**Published:** 2017-03-26

**Authors:** Anna Delprato

**Affiliations:** BioScience Project, Wakefield, USA

We thank the reviewer for the insightful comments and suggestions. Below we respond to each point of the review. We have submitted an updated version of the article that incorporates the requested changes and provides additional information about the internship program.

***Reviewer’s comment***

*1) The authors stipulate in the abstract that the students in the program were "capable of comprehending ...such a project". The authors cannot make that claim with the evidence presented in the manuscript. Self-reported surveys were the only form of data presented, and this type of survey does not show student learning or comprehension. The reader also does not know if this comprehension is applied to the ability to perform research, the content learned, or learning how to communicate the information (or a combination of all three). This is a significant concern and must be addressed before publication.*

**Author’s response**

 We have removed the statement that students comprehended the internship project from the abstract as this was not formally evaluated in the pilot study.

***Reviewer’s comment***

*2) There were only six references cited for the entire manuscript. There are many papers in the literature that discuss programs similar in nature to this. It would behoove the authors to include more background research to support their claims.*

**Author’s response**

 We have provided additional references as requested. If the reviewer is aware of other relevant references, we will include them in the article.

***Reviewer’s comment***

*3) The authors need to include more information about the project itself (i.e. a discussion of the modules used for teaching, the mentoring, etc.). The reader is left wondering if the students are doing actually self-initiated projects, or if they are being led by the modules.*

*It was helpful to read about the databases that the students use, but the reader does not gain any knowledge about the actual e-internship program through which the students progress (or are those the modules? It is unclear.) How are the modules designed? Is there any research to show that the module design is effective?*

**Author’s response**

 The modules are based on a protocol that was designed in our laboratory for profiling gene expression data to identify genes of interest associated with a behavior or brain disorder. The protocol is broken down into steps so as not to overwhelm the students with too much information at once. Modules which consist of detailed instruction are built around each step of the protocol. The purpose of the module is to provide students with a detailed reference in addition to the mentoring sessions so that they can work in the databases and with the data independently.

 The effectiveness of the modules has not been formally evaluated.

 We would like to also provide the students with screen casting videos for improved instruction. We hope to make the videos available to students this season.

***Reviewer’s comment***

*4) In other words, how were they mentored? Is the mentoring effective for student growth?*

**Author’s response**

 Student mentoring primarily occurs through video conferencing and includes a walk-through of each database using screen sharing, project/topic discussion, and troubleshooting. Mentoring sessions occur weekly and last anywhere from 30 min - 90 min. Students also email us with questions.  

 The effectiveness of the mentoring sessions on student growth has not been assessed formally but we believe that mentoring is necessary for the students to perform their research and complete their projects.

***Reviewer’s comment***

*5) Do they present and defend their research in a scientific forum (even online)?*

**Author’s response**

 Students have not presented and/or defended their research in a scientific forum, but this is a great idea and we will add it to this year’s program.

***Reviewer’s comment***

*6) It would also help to put the Project Design section first (before the database discussion).*

**Author’s response**

 Done

***Reviewer’s comment***

*7) The evaluation of the program needs to be more robust. Self-reported surveys are a good starting point, but they are only formative assessments. You need to include some summative assessments for the program and more qualitative assessments to determine the efficacy of the program for student learning.*

*Also, it sounds as though the students are spending much less time on the project as opposed to an onsite laboratory model, so the reader is left wondering if the program is really effective.*

**Author’s response**

 The internship described in this study constitutes a dry lab experience. All of the research and analysis is done on a computer. Performing this type of work for 6 to 8 hours per day is physically and mentally taxing and can result in errors. We recommend that students spend about 10 to 15 hours per week working on their projects. The nature of bioinformatics-based research is very different from a classical wet lab experience which entails physical manipulations at the bench. We believe that both types of research internships are valuable to students but program efficacy cannot be evaluated based solely on time spent given the differences between the two approaches.

***Reviewer’s comment***

*8) Also, do you have demographics of the participating students?*

**Author’s response**

 We ask students to provide us with their resume in order to get an idea of their science background but we do not collect demographic information.

***Reviewer’s comment***

*9) There are a couple of grammatical errors (mostly words that need to be omitted and comma usage errors).*

**Author’s response**

 Corrected

***Reviewer’s comment***

*10) In summary, this manuscript presents an interesting program, but the authors need to address the issues of a clear evaluation plan to determine the efficacy of the program, and provide enough discussion of the program so that readers understand exactly what the students gain.*

# Peer review: Referee response for version 1

**DOI:** 10.5256/f1000research.11391.r19553

**Published:** 2017-02-08

**Authors:** Byron C. Jones

**Affiliations:** 1 Department of Genetics, Genomics, and Informatics, University of Tennessee Health Science Center, Memphis, TN, USA

The description of the internship program is clear and convincing. One minor issue is with Table 2 -- the ** is missing from the body of the table.

 Are there plans to obtain financial support for the students?

I have read this submission. I believe that I have an appropriate level of expertise to confirm that it is of an acceptable scientific standard.

# Response

**Published:** 2017-02-21

**Authors:** Anna Delprato

**Affiliations:** BioScience Project, Wakefield, USA

Thank you for reviewing our article. We are seeking funding for student support and program expansion.

# Peer review: Referee response for version 1

**DOI:** 10.5256/f1000research.11391.r19850

**Published:** 2017-02-08

**Authors:** Christine F. Hohmann

**Affiliations:** 1 Department of Biology and RISE Program, Morgan State University, Baltimore, MD, USA

The paper by Crusio, Rubino and Delprato, appropriately titled: **“Engaging high school students in systems biology through an e-internship program”,** provides a description of a research education program at the high school level. The authors have developed an online research training program that uses public access genomics and neuroscience data bases to train students in behavioral neurogenetics. Students exercise a substantial amount of autonomy (“are able to choose their own projects”, “proceed at their own pace”). Students were mentored through the process of identifying their projects, conducting their research and preparing a capstone poster presentation by the co-author, Dr. Anna Delprato. According to the website for the program, all students who participated in the project successfully submitted their capstone posters which appear to be of a very sophisticated nature. One student even accomplished to co-author a research paper with the first author of this paper. Unfortunately, the viewing option online (at least for this reviewer and her mac computer; maybe I need tech help??) made it impossible to read the *details* of the posters and after download, the image quality was insufficient, when enlarged, to see much. Survey data indicate that the participants were, with few exceptions in year 1, very satisfied with this learning opportunity which had two iterations so far.

 This online training/mentoring model offers a very exciting possibility for (global) distance learning. It is currently based on a very small students sample (5 year one and 10 in year 2) from just a couple of high schools located in New England and on limited assessment. It would have been helpful to know the demographics of the student population involved as well as the graduation rates at this school and how many graduates typically attend college. It would also be helpful to know how many hours, on average, Dr. Delprato spend with each student/student group over the course of the summer. I hope that, as the authors continue their model, they will follow their participants’ future educational and career decisions to assess the impact of the training experience. There is a lot of potential in this model to be implemented within the context of course based research, at the college level as well as for integration into federally funded existing training programs in the US. I would like to encourage the authors to prepare a publication on the specifics of their curriculum in near future, if they are interested in having others adopt this model and implement it in different settings.

 In conclusion, although this is a very preliminary and descriptive account of a summer research training experience its novelty merits publication even at this early stage. The paper provides sufficient detail to engender ideas for others to attempt to replicate the model, although a more detailed description of the curriculum, and follow up analysis with a larger data set, should be encouraged.

I have read this submission. I believe that I have an appropriate level of expertise to confirm that it is of an acceptable scientific standard.

# Response

**Published:** 2017-02-21

**Authors:** Anna Delprato

**Affiliations:** BioScience Project, Wakefield, USA

Thank you for reviewing our article. We have changed the file format of the students posters to pdf. This has improved the resolution for online viewing.
